# Interventions to improve medication adherence in tuberculosis patients: a systematic review of randomized controlled studies

**DOI:** 10.1038/s41533-020-0179-x

**Published:** 2020-05-11

**Authors:** Ivan S. Pradipta, Daphne Houtsma, Job F. M. van Boven, Jan-Willem C. Alffenaar, Eelko Hak

**Affiliations:** 1 Unit of Pharmaco-Therapy, -Epidemiology and -Economics (PTE2), Groningen Research Institute of Pharmacy, University of Groningen, Groningen, the Netherlands; 2 Department of Pharmacology and Clinical Pharmacy, Faculty of Pharmacy, Universitas Padjadjaran, Jawa Barat, Indonesia; 3 Center of Excellence in Higher Education for Pharmaceutical Care Innovation, Universitas Padjadjaran, Jawa Barat, Indonesia; 4 Department of Clinical Pharmacy and Pharmacology, University Medical Center Groningen, University of Groningen, Groningen, the Netherlands; 5 Medication Adherence Expertise Center of the northern Netherlands (MAECON), Groningen, the Netherlands; 6 Faculty of Medicine and Health, School of Pharmacy, University of Sydney, Sydney, NSW Australia; 7 Westmead Hospital, Sydney, NSW Australia; 8 Marie Bashir Institute for Infectious Diseases and Biosecurity, Sydney, NSW Australia

**Keywords:** Outcomes research, Epidemiology, Clinical trial design

## Abstract

Non-adherence to anti-tuberculosis (anti-TB) medication is a major risk factor for poor treatment outcomes. We therefore assessed the effectiveness of medication adherence enhancing interventions in TB patients. We report a systematic review of randomized controlled trials that included either latent tuberculosis infection (LTBI) or active TB patients. Outcomes of interest included adherence rate, completed treatment, defaulted treatment and treatment outcomes. We identified four LTBI and ten active TB studies. In active TB patients, directly observed treatment (DOT) by trained community workers, short messaging service combined with education, counselling, monthly TB vouchers, drug box reminders and combinations of those were found effective. In LTBI patients, shorter regimens and DOT effectively improved treatment completion. Interestingly, DOT showed variable effectiveness, highlighting that implementation, population and setting may play important roles. Since non-adherence factors are patient-specific, personalized interventions are required to enhance the impact of a programme to improve medication adherence in TB patients.

## Introduction

Tuberculosis (TB) remains an important worldwide health issue. World Health Organization (WHO) reported that TB is the cause of illness for around 10 million people every year and has been ranked among the top ten causes of death globally^1^. TB, caused by *Mycobacterium tuberculosis*, can be spread easily from patients suffering from pulmonary TB to healthy people by air transmission^1^. Consequently, anti-TB drug treatment is required for TB patients to cure the disease and prevent disease transmission.

Comparable to other complex diseases, TB patients have to be treated with several drugs for a long period. According to the WHO guideline, active pulmonary TB patients should take drugs for at least 6 months^2^, while latent tuberculosis infection (LTBI) patients should take drugs for at least 3 months^3^. The treatment duration can be extended if TB patients are diagnosed as multi-drug resistant tuberculosis (MDR-TB), a resistance of the pathogen to the most potent anti-TB medicines (isoniazid and rifampicin). MDR-TB treatment can be up to 24 months using multiple drugs^4^.

Poor adherence to medication is widely known as a causal factor for increased risk of morbidity, mortality and cost burden^5–7^. A global meta-analysis revealed that non-adherence to treatment is a risk factor for MDR-TB^8^. Furthermore, MDR-TB patients, as compared to drug-susceptible patients, have more frequently poor treatment outcomes^9,10^. Treatment adherence is affected by multiple factors. These factors are divided into five different interacting dimensions, including socio-economic, health care system, condition, therapy and patient factors^11^. Although studies on adherence in other diseases than TB showed that interventions targeting these factors can significantly improve adherence rates^12–14^, a better understanding of the effects of possible interventions in TB is required. We therefore systematically reviewed the effectiveness of various interventions to improve medication adherence in LTBI and active TB patients.

## Results

### Study selection

**Fig. 1:**
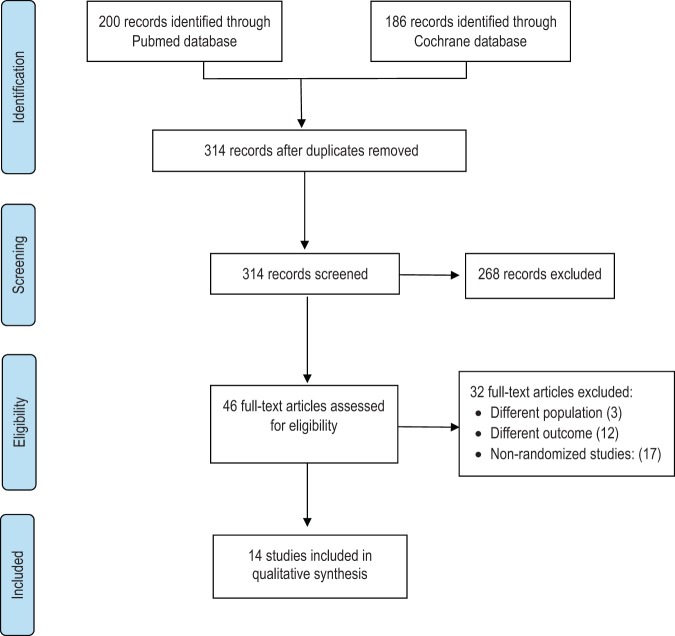
Flow diagram of the included articles. The PRISMA flowchart reporting the number of papers identified, screened, excluded and included.

During the search, we found 200 records from the Medline/PubMed database and 186 records from the Cochrane database. We identified 72 duplicate records using the Refwork® software. A total of 314 articles were screened for the title and abstract. This initial screening excluded 268 irrelevant records, then the full-text screening process was continued for 46 records. In the full-text screening, 32 articles were excluded owing to different populations (3 articles), different study outcomes (12 articles) and non-randomized study design (17 articles). We finally analysed 14 studies for qualitative synthesis. The flow diagram, literature search and screening process are presented in Fig. 1.

### Study characteristics and interventions

In total, 15,507 subjects were included in the analysis. In all, 1991 LTBI patients and 13,516 active TB patients participated. The minimum number of subjects in the included studies was 89, while the maximum number was 4154 subjects. The included studies were conducted in both low- and high-burden TB countries, i.e. Pakistan^15,16^, Australia^17^, Iraq^18^, China^19,20^, Senegal^21^, South Africa^22–24^, Timor Leste^25^, Canada^26^, United States^24,27^, Spain^24^, Hong Kong^24^ and Mexico^28^.

The 14 randomized controlled trials (RCTs) assessed a broad range of adherence management interventions. These included Short Message Service (SMS) intervention^15,16,18–20,24^, directly observed treatment (DOT) administered by health care workers^24^, family members^17^ and non-health care worker communities^18,22^, a reinforced counselling method^21^, a trained lay health workers intervention to manage TB case^22^, monthly TB vouchers^23^, a drug box reminder^20^, a combination text messaging and drug box reminder^20^, a nutritious intervention^25^, shorter regimen^26^, a peer-based intervention^27^ and a behavioural intervention^28^. The characteristics of the included studies are shown in Table 1.

**Table 1:** Characteristics of the included articles.

Authors	Study design	Study period	Type of participant	Setting	Intervention	Control	Outcomes
Mohammed et al.^15^	RCT	2011–2014	Adult newly TB patients	TB clinics and hospitals in Karachi, Pakistan	Zindagi SMS, a two-way SMS reminder system, sent daily SMS reminders and motivational messages to participants and asked them to respond through SMS or missed calls after taking their medication	Standard of care	Treatment completion (sum of completed treatment and cured treatment) and defaulted treatment
MacIntyre et al.^17^	RCT	1998–2000	Adult newly TB patients	Two clinics in the North-Western Health care network, Victoria, Australia	DOT administered by a family member	Standard supervised but non-directly observed treatment	Treatment completion (sum of completed treatment and cured treatment)
Mohan et al.^18^	RCT	2001	Adult newly TB patients	15 TB centres in Baghdad, Iraq	DOT and home visits from trained members of the Iraqi Women’s Federation	DOT without home visits	Cured and defaulted treatment; sputum conversion: a negative sputum smear at the fifth month after treatment
Fang et al.^19^	RCT	2014–2015	Adult pulmonary TB patients	Six districts in Anhui Province, China	Regular SMS to remind taking medicine and educate core knowledge about pulmonary TB. SMS contents: (a) following the doctor’s instructions and taking medicine timely, (b) re-examining sputum and chest X-ray periodically, (c) covering nose and mouth when sneezing or coughing and not spitting everywhere, (d) paying attention to washing hands, opening a ventilated window regularly, doing sports more, and improving resistibility, (e) adhering to regular treatment, and most of TB patients can be cured	DOT	Treatment completion (sum of completed treatment and cured treatment) and sputum conversion: a negative sputum smear at the sixth month after treatment
Thiam et al.^21^	Cluster RCT	2003–2005	Newly diagnosed TB patients	Government district health centres in Senegal	Reinforced counselling through improved communication between health personnel and patients, decentralization of treatment, choice of DOT supporter by the patient and reinforcement of supervision activities	The usual standard care of TB	Cured and defaulted treatment
Clarke et al.^22^	Cluster RCT	2000–2001	Adult newly TB patients	Farms in the Boland health district, Western Cape, South Africa	Adult farm dwellers selected as trained lay health workers to screen, refer, report, educate, motivate and observe the treatment of TB patients	No intervention of lay health workers	Treatment completion (sum of completed treatment and cured treatment)
Farooqi et al.^16^	RCT	2014–2015	Adult newly pulmonary and extra-pulmonary TB patients	Khyber Teaching Hospital Peshawar and Emergency Satellite Hospital Nahaqi, Pakistan	DOT and daily mobile SMS reminders	DOT	Treatment completion (sum of completed treatment and cured treatment) and defaulted treatment
Lutge et al.^23^	Cluster RCT	2009–2010	Adult newly TB patients	Primary public health care at Kwazulu-Natal, South of Africa	Monthly voucher USD 15 per month	No monthly voucher	Treatment completion (sum of completed treatment and cured treatment) and defaulted treatment
Liu et al.^20^	Cluster RCT	2009	Adult newly pulmonary TB patients	Provinces of Heilongjiang, Jiangsu, Hunan and Chongqing, China	Text messaging reminder	The usual care: treatment monitoring can be self-administered treatment or treatment supervised by family members or treatment supervised by health care workers. The local doctor monitor the treatment	Poor adherence was due to the percentage of patient-months where at least 20% of doses (15 doses) were missed; poor treatment outcome
					Drug box reminder		
					Combined (text messaging and drug box reminder)		
Martins et al.^25^	RCT	2005–2006	Adult newly pulmonary TB patients	Three primary care clinics in Dili, Timor-Leste	Nutritious, culturally appropriate daily meal (weeks 1–8) and food package (weeks 9–32)	Nutritional advice	Treatment completion: clearance of acid-fast bacilli from the sputum after treatment or the completion of 8 months of treatment or both; adherence to treatment: clinic attendance, DOT, interview and pill count
Menzies et al.^26^	RCT	2002	Adult LTBI	A university-affiliated respiratory hospital, Canada	4 months of daily rifampicin 10 mg/kg	9 months of daily isoniazid 5 mg/ kg	Completed treatment defined as ≥80% took doses within 20 weeks for 4RIF or within 43 weeks for 9INH
Belknap et al.^24^	Non-inferiority RCT	2012–2014	Adult LTBI patients	Outpatient tuberculosis clinics in the United States (9 sites), Spain (1 site), Hong Kong (1 site) and South Africa (1 site)	Directly observed treatment	SAT with monthly monitoring	Treatment completion (sum of completed treatment and cured treatment)
					SAT with monthly monitoring and text message reminders		
Hirsch-Moverman et al.^27^	RCT	2002–2005	Adult LTBI patients	The Harlem Hospital Chest Clinic in New York, NY, USA	Peer-based intervention: peers educated and coached patients on adherence; gave social and emotional support and provided health care and social service system navigation, together with patients and health workers, to enhance patient–provider communication. The peers were people who had completed LTBI or anti-TB treatments and had attended a 4-week training programme that includes role-playing exercise, informational sessions and observation. Peers met participants by one-on-one at least once a week	Self-administered 9-month isoniazid treatment	Treatment completion (sum of completed treatment and cured treatment)
Hovell et al.^28^	RCT	1996–2000	Adolescent LTBI patients	San Diego-Tijuana, Mexico–United States	Usual care plus adherence coaching: monthly case review and discussion about adherence problems and advice	The usual medical care: 300 mg INH per day was prescribed for 6–9 months with monthly evaluation	Treatment completion: completion of LTBI treatment as taking 180 pills within 270 days
					Usual care plus self-esteem counselling: monthly meeting about relationship and communication with family, friends and cultural identity to enhance self-esteem		

*RCT* randomized controlled trial, *LTBI* latent tuberculosis infection, *DOT* directly observed treatment, *SAT* self-administration therapy, *SMS* Short Message Service, *AFB* acid-fast bacilli.

### Study outcomes

In the active TB patients, the primary outcomes were treatment completion^15–17,19,22,23^, interrupted rate^15,16,18,21,23^ and adherence rate^20,25^, while the other outcomes were negative sputum conversion^18,19^, cured^18,21^ and poor treatment outcomes^20^. In the LTBI studies, we observed that treatment completion was the only outcome. Regarding the intervention effect, not all interventions significantly improved drug adherence and treatment outcomes. Several interventions were found effective in improving medication adherence and outcomes of active TB patients, i.e. DOT by trained community members, SMS combined with TB education, a reinforced counselling method, monthly TB voucher, drug box reminder and a combination drug box reminder and text messaging. However, only two studies reported adherence rate as the study outcome^20,25^. We identified that a drug box reminder (mean ratio (MR) 0.58; 95% confidence interval (CI) 0.42 to 0.79) and its combination with text messaging (MR 0.49; 95% CI 0.27 to 0.88) significantly reduced missing a drug dose among active TB patients^20^, while food incentives were not significantly different from the comparator for the intensive (MR −4.7; 95% CI −0.8 to −8.6) and continuation phase (MR 0; 95%CI −1.7 to 1.7) in the active TB patients^25^, see Table 2.

**Table 2:** Effect of the intervention on the study outcomes in active tuberculosis patients.

No.	Authors	Intervention	Intervention target	Number of participants		Study outcomes					
				Intervention group	Comparator group	Treatment completion	Interrupted rate	Adherence rate	Cured treatment	Sputum conversion	Poor treatment
1	Mohammed et al.^15^	Two-way SMS reminder system with motivational words	Patient and health care	1104	1093	RR 1.00; (0.96 to 1.04)	RR 0.92; (0.68 to 1.24)	—	—	—	—
2	MacIntyre et al.^17^	Family DOT	Health care	87	86	RR 1.96; (0.98 to 1.15)	—	—	—	—	—
3	Mohan et al.^18^	DOT and home visits by trained members of the Iraqi Women’s Federation	Patient and health care	240	240	—	RR 0.83; (0.02 to 0.34)	—	RR 1.2; (1.14 to 1.33)	RR 1.26; (1.15 to 1.37)^a^	—
4	Fang et al.^19^	SMS and regular education of core knowledge about pulmonary TB	Patient and health care	160	190	RR 1.11; (1.04 to 1.18)	—	—	—	RR 1.26; (1.14 to 1.42)^b^	—
5	Thiam et al.^21^	Reinforced counselling method	Patient and health care	778	744	—	RR 0.43; (0.21 to 0.89)	—	RR 1.18; (1.03 to 1.34)	—	—
6	Clarke et al.^22^	Trained LHWs intervention	Patient and health care	47	42	RR 1.08; (0.92 to 1.27)	—	—	—	—	—
7	Farooqi et al.^16^	Mobile SMS reminders	Patient and health care	74	74	RR 1.01; (0.95 to 1.10)	RR 0.75; (0.17 to 3.24)	—	—	—	—
8	Lutge et al.^23^	Monthly voucher USD 15 per month	Socio-economy	2170	1984	RR 1.07; (1.04 to 1.11)	0.06 (0.04 to 0.11)	—	—	—	—
9	Liu et al.^20^	Text messaging reminder	Patient and health care	996	1091	—	—	MR 0.94 (0.71 to 1.24)	—	—	MR 0.44 (0.17, 1.13)
		Drug box reminder		992		—	—	MR 0.58 (0.42 to 0.79)	—	—	MR 0.71 (0.33 to 1.51)
		Combination of text messaging and drug box reminder		1059		—	—	MR 0.49 (0.27 to 0.88)	—	—	MR 1.00 (0.45 to 2.20)
10	Martins et al.^25^	Food incentive	Patient	136	129	RR 0.98 (0.86 to 1.11)	—	MR -4.7 (−0.8 to −8.6)^a^ MR 0 (−1.7 to 1.7)^c^	—	—	—

Treatment completion is completing the prescribed doses of drugs; interrupted treatment is a defaulted or/and interrupted treatment groups that were compared with non-interrupted patient group; poor treatment is a combination of defaulted, failed treatment and death outcome; adherence rate is a proportion of missing anti-TB drug dose; sputum conversion is a conversion sputum to a negative result.

*MR* mean ratio, *RR* relative risk, *OR* odds ratio, *TB* tuberculosis, *LTBI* latent tuberculosis infection, *DOT* directly observed treatment, *SAT* self-administration therapy, *SMS* Short Message Service, *AFB* acid-fast bacilli, *USD* United States Dollar, *LHW* lay health worker.

^a^Intensive phase.

^b^Conversion rate in the sixth month of treatment.

^c^Continuation phase.

In the LTBI patients, shorter regimens and DOT interventions significantly improved treatment completion. We identified that 4 months of daily rifampicin 10 mg/kg was more effective to improve treatment completion than 9 months of daily isoniazid 5 mg/kg (relative risk (RR) 1.2; 95% CI 1.02–1.4)^26^, while DOT intervention was more effective to improve treatment completion than self-administration therapy (SAT) with monthly monitoring (RR 1.18; 95% CI 1.09–1.27)^24^. In contrast, several interventions such as SAT with weekly text message reminders plus monthly monitoring (RR 1.03; 95% CI 0.95–1.13)^24^, a peer-based intervention (RR 1.06; 95% CI 0.86–1.31)^27^, adherence coaching intervention (RR 1.36; 95% CI 0.98–1.88)^28^ and self-esteem counselling (RR 1.12; 95% CI 0.78–1.58)^28^ did not significantly improve treatment completion in LTBI patients, see Table 3.

**Table 3:** Effect of the interventions on the study outcomes in latent tuberculosis infection (LTBI) patients.

No.	Authors	Intervention	Intervention target	Number of participants		Study outcome
				Intervention group	Comparator group	Treatment completion
1	Menzies et al.^26^	4 month of daily rifampicin 10 mg/kg	Treatment	58	58	RR 1.2; (1.02–1.4)
2	Belknap et al.^24^	DOT	Patient and health care	337	337	RR 1.18; (1.09–1.27)
		Self-administration therapy with weekly text message reminders and monthly monitoring	Patient and health care	328	337	RR 1.03; (0.95–1.13)
3	Hirsch-Moverman et al.^27^	Peer-based intervention	Patient and health care	128	122	RR 1.06; (0.86–1.31)
4	Hovell et al.^28^	Usual care plus adherence coaching	Patient and health care	92	96	RR 1.36; (0.98–1.88)
		Usual care plus self-esteem counselling	Patient and health care	98		RR 1.12; (0.78–1.58)

Treatment completion is completing the prescribed doses of drugs.

*DOT* directly observed treatment, *RR* relative risk.

### Quality assessment of the included studies

A double-blinding method was either not possible or not applied for most of the included studies. This is due to the fact that intervention activities were impossible to blind, such as SMS reminders, DOT, counselling, monthly vouchers, drug box reminders, food incentive and peer-based intervention. Often, an open-label design was applied. Therefore, none of the included had a maximum JADAD score (five points). Three points of the JADAD score was the maximum score among the included studies because none of the studies applied the blinding method. Five of the 14 studies had the lowest JADAD score (2 points) due to the absence of a description of randomization procedure^17–19,27,28^. The other studies had a higher score (three points) given they appropriately described the randomization method and clearly illustrated the withdrawals of participants^15,16,20–26^. The risk of bias assessment is presented in Table 4.

**Table 4:** Risk of bias assessment for randomized studies using the JADAD score.

No.	Author	Randomization	Description of randomization	Double-blind method	Description of the blinding method	Description of participant withdrawal/drop-out	Total score
1	Mohammed et al.^15^	1	1	0	0	1	3
2	MacIntyre et al.^17^	1	0	0	0	1	2
3	Mohan et al.^18^	1	0	0	0	1	2
4	Belknap et al.^24^	1	1	0	0	1	3
5	Fang et al.^19^	1	0	0	0	1	2
6	Hirsch-Moverman et al.^27^	1	0	0	0	1	2
7	Thiam et al.^21^	1	1	0	0	1	3
8	Clarke et al.^22^	1	1	0	0	1	3
9	Farooqi et al.^16^	1	1	0	0	1	3
10	Hovell et al.^28^	1	0	0	0	1	2
11	Lutge et al.^23^	1	1	0	0	1	3
12	Menzies et al.^26^	1	1	0	0	1	3
13	Liu et al.^20^	1	1	0	0	1	3
14	Martins et al.^25^	1	1	0	0	1	3

The JADAD questions: (1) Was the study described as randomized?; (2) Was the method used to generate sequence of randomization described and appropriate?; (3) Was the study described as double blind?; (4) Was the method of double-blinding described and appropriate?; (5) Was there a description of withdrawals and dropouts? A double-blinding method was either not possible or not applied for the included studies.

## Discussion

We observed various interventions that were successful in improving medication adherence and outcomes in TB patients. The interventions targeted several factors of adherence, such as socio-economic, patient, health care and treatment aspects. The effective interventions to improve treatment completion in active TB patients were DOT with daily home visits by community-trained members, SMS reminders combined with TB education, a reinforced counselling method and a monthly voucher intervention. In LTBI patients, DOT and a shorter regimen significantly improved treatment completion. We identified that the drug box reminder or its combination with text messaging reminders significantly improved medication adherence rates among active TB patients, while no studies were found showing an effective intervention to improve medication adherence rate in LTBI patients. In contrast, we found that some interventions, such as SMS reminders or its combination with motivational messages, family DOT, involving trained lay health workers in TB management, and food incentives were not significantly different compared with the comparator groups regarding improving treatment completion and outcomes in active TB patients. Similarly, we identified SMS reminders combined with monthly monitoring, peer-based intervention, coaching adherence and self-esteem counselling were not effective in improving treatment completion in LTBI patients.

Interestingly, interventions using DOT showed variable effects on the study outcomes. Family DOT^17^ were not superior in the improvement of treatment outcomes among active TB patients, while institutional DOT significantly improved treatment completion in LTBI patients. Unfortunately, a meta-analysis of the crude data was impossible, because heterogeneities were identified across the included studies with regard to population, intervention and study outcomes. However, a previous meta-analysis stated that the understanding of resources and situations in which DOT can be beneficial is an essential part of successful implementation of DOT^29^. Furthermore, the interaction between DOT providers and TB patients may also influence the effect on medication adherence and outcome. Therefore, the differences of resources, situation and interaction between DOT providers and TB patients may indicate that the effects of interventions can vary across studies and settings.

Generally, the differences in observed effect sizes of the interventions in this review can be explained by several aspects: (1) characteristics of the subjects, (2) measurement method of the adherence (outcome), (3) characteristics of the comparator group, and (4) the quality of the study design and intervention. According to WHO^11^, adherence is a multidimensional phenomenon that can be determined by the interaction of the five essential factors, i.e., socio-economic, provider–patient/health care system, condition related, therapy related and patient related. Since the essential causal factors for poor adherence can be individual, assessing the individual non-adherence factors is a critical approach to have effective personalized interventions to increase medication adherence. The most optimal intervention to improve medication adherence should not be “one-size-fits-all”. As an example, an intervention using SMS reminders for taking medicine may not be effective if the individual problem of medication adherence is mainly caused by inaccessibility of the patient to have a qualified medicine.

In terms of outcome measurement, heterogeneity was shown in the included studies. Most of the studies used a treatment completion parameter measured by temporary patient visits or self-reported/medical documentation as the outcome parameter for medication adherence. An implication of this is the possibility of misclassification of medication adherence. The measurement did not represent the daily consumption of the medicines during the treatment phase. Hence, potential information bias is high in the studies that used temporary patient visits or self-reported/medical documentation to assess medication adherence. The accuracy of adherence measurement in TB patients was reported in a systematic review^30^. Methods to measure adherence can be categorized as direct (e.g. DOT, ingestible sensors, drug or metabolites measurements) and indirect (e.g. patient self-report, pill counts, health information system, electronic pill bottles and SMS). Currently, digital adherence technologies have been developed that offer large potential to measure and improve medication adherence in TB patients^31^. The technology potentially facilitates monitoring of adherence that provides a more patient-centric approach than the existing DOT^32^. The digital technology for monitoring medication adherence of TB patients was reported in the form of video observed therapy and electronic medication monitoring^33^. Measurement of medication adherence was also reported using pharmaceutical databases^33^. However, considering the accuracy and validity, direct measurement should be preferably used for measuring medication adherence in an interventional study.

There were some variations in the comparator group in the included studies, which may also have affected the validity of the findings. We noted that self-administration of treatment without supervision and DOT were the comparator groups in the included studies. Theoretically, the effect of the studied intervention will be higher in the studies that used self-administration without supervision as the comparator group instead of DOT. A previous study showed that DOT was more effective than SAT in the improvement of treatment adherence^34^ and DOT was also recommended by WHO for improving treatment adherence in TB patients^34^. It is possible, therefore, that using different comparators to compare two or more intervention studies will lead to an under- or over-estimation.

Another aspect, which may explain the variations in the results of studied interventions, is the quality of the included studies. Among the randomized studies, the randomization method was unclear in five studies^17–19,27,28^. The investigators did not describe how the random allocation was conducted. In most of the included studies, blinding was impossible. Since the intervention involved direct activities with the research subjects such as reminders, counselling, education and incentives, performing a blinding procedure was impossible. In addition, the quality of implementation of the intervention is also essential. For instance, in DOT studies, the ability of the treatment observer to improve medication adherence of TB patients will affect the success of the intervention. As previously described, the interaction between treatment observer and TB patients should therefore be considered in order to understand potential changes in medication adherence.

Several limitations to our review should be acknowledged. First, the review was based on the two databases with restriction to English publications and searching period, hence not all the intervention studies may be covered in this study. However, to the best of our knowledge, the vast majority of relevant studies are published in the English language and recent trials incorporated knowledge from the potential trials before 2003. Second, only a few studies used adherence rate as the study outcome. Most of the studies mentioned treatment outcomes (i.e. sputum conversion, cured and poor treatment outcome) but did not include sufficient detail on medication adherence as the study outcome. Since treatment outcomes were associated with medication adherence in previous studies^5,6,8^, we included treatment outcomes as the secondary outcomes in this review. Therefore, the effectiveness of the interventions to improve medication adherence, as reported in the studies, should be carefully interpreted, and clearly high-quality intervention studies should be developed in the future. Lastly, in order to assess intervention effects in a homogeneous population regarding patient characteristics, this review excluded interventions in more complex or high-risk TB patients, such as those with comorbid human immunodeficiency virus (HIV), drug-resistant TB, alcoholism and illicit drug use. We acknowledge that these are, however, important subgroups regarding non-adherence, for which we recommend separate, focussed studies and reviews.

Our review highlighted various potential interventions to improve medication adherence among LTBI and active TB patients. Characteristics of the research subjects, accurate measurement of the adherence, type of the comparator group, the robustness of study design and implementation of the intervention should be considered to observe an effective and unbiased intervention for medication adherence in TB patients. Since non-adherence factors can be individual, interventions that takes into account individual patient barriers are required to have an effective medication adherence programme. Therefore, future intervention studies should use objective adherence measures and focus on the effectiveness of TB medication adherence programmes that use a more personalized approach.

## Methods

### Literature review

We performed a systematic review of articles that were published between January 1, 2003 and April 24, 2018 and reported in the English language. According to the study protocol, the search period was restricted to articles published from 2003 onwards because in that year the influential WHO Adherence report was published and created wide-scale awareness on the issue of non-adherence ever since^11^. This systematic review was reported according to Preferred Reporting Items for Systematic Reviews and Meta-Analyses guidance^35^. The PICOS items, i.e. population, intervention, comparator, outcomes and study design, are specified in the following sections.

### Population

To be able to better distinguish between the potential impact of experienced symptoms on extent of adherence, the study population was divided into two different groups, i.e. patients with LTBI and active TB. The status of TB disease should be confirmed by clinical or laboratory examination (e.g. TB symptoms, Mantoux, IGRA, chest radiograph or other TB examination) and/or microbiological verification (e.g. smear sputum test, phenotyping drug susceptibility test or polymerase chain reaction). In order to draw conclusions based on a population as homogeneous as possible, we excluded studies restricted to specific high-risk treatment non-adherence groups, such as TB patients with HIV, drug-resistant TB, alcoholism and illicit drug use.

### Interventions and comparator

Studies that analysed interventions related to improving medication adherence and treatment outcomes were included in this review. The intervention was allowed to target one or multiple factors of adherence, such as socio-economic, health care team and system, health condition, therapy or patient factors. The intervention should have a comparison group to analyse the effect of the intervention.

### Outcomes

In terms of the study outcomes, we followed the global definition published by WHO in 2014^36^. We defined medication adherence as the primary outcome. Of note, medication adherence consists of three phases: initiation, implementation, and persistence^37^. In our assessment, persistence was deemed a synonym for “completed treatment” and non-persistence was a synonym for “defaulted treatment”. Implementation was deemed similar to “adherence rate”. Moreover, we defined “cured treatment”, “negative sputum conversion” and “poor treatment” outcomes as the secondary outcome in this study.

According to the global definition, “completed treatment” was defined as a TB patient who completed treatment without evidence of failure but with no record showing that sputum smear or culture results were positive in the last month of treatment, while “defaulted treatment” was defined as an interruption of TB treatment for two or more consecutive months. “Adherence rate” was identified by the proportion of anti-TB drug dose taken during the treatment period. As the other outcomes, “cured treatment” was defined as smear or culture negative in the last month of treatment and on at least one previous occasion, while “negative sputum conversion” was defined as the conversion sputum to a negative result. Furthermore, “poor treatment outcome” is a combination of defaulted, failed treatment and death outcome. Failed treatment was defined as a positive sputum smear or culture at the fifth month after treatment initiation.

### Study design

We only included RCTs. Given their increased risk for bias, quasi-experimental, cohort, cross-sectional, case–control, case reports, case series, review articles and abstract conference were not eligible for inclusion.

### Data collection

The relevant articles were obtained from the Medline/PubMed and Cochrane databases with specific key terms. To effectively obtain the relevant articles, we used restriction to the following filters in the Medline/PubMed database, such as clinical trial, comparative study, controlled clinical trial, observational study, RCT and humans. Applying observational study in the Medline/PubMed filter was intended to anticipate potential RCTs in the group labelled as observational studies. Key terms for obtaining the articles can be found in Supplementary Information.

### Data extraction and quality assessment

Title and abstract of the articles were screened by I.S.P. and D.H., then the full-text of the articles were assessed for the eligibility and quality by I.S.P. Duplicated articles from two databases were removed using the Refwork^®^ software. The eligible articles were then reviewed for relevant information. Information related to year of publication, population, type of intervention, comparator group and study outcome was extracted by I.S.P. Any disagreements between the reviewers during the screening phase were solved by discussion until consensus was reached.

Regarding the quality assessment, we used the JADAD score for assessing the quality of the RCTs^38^. Three main domains were appraised in the JADAD score system, i.e. randomization, blinding method and subject withdrawal. The domains were assessed by five questions. For each question, a study could earn one point, with a total score of five points. The five questions are described as follows: Was the study described as randomized?; Was the method used to generate sequence of randomization described and appropriate?; Was the study described as double blind?; Was the method of double-blinding appropriately described?; and Was there a description of withdrawals and dropouts?

### Summary measures and synthesis of results

The total number and group of patients with any specific outcome for both primary and secondary outcomes were extracted by I.S.P. and summarized in tables. For the point estimate of the intervention, we used RR for dichotomous outcome data and MR for continuous outcome data with a 95% CI.

### Reporting summary

Further information on research design is available in the Nature Research Reporting Summary linked to this article.

## Supplementary information

Supplementary Information

Reporting Summary

## Data Availability

The data that support the findings of this study are publicly available through the following electronic biomedical literature databases: Medline/PubMed and Cochrane.

## Code availability

No special code was described in this report.
