# Supplementary material for: Interventions to improve medication adherence in tuberculosis patients: a systematic review of randomized controlled studies
Source: NPJ Prim Care Respir Med. 2020 May 11;30:21. doi: 10.1038/s41533-020-0179-x (PMC7214451; doi:10.1038/s41533-020-0179-x)
Supplement: Supplementary file 1 — Supplementary Information [file 41533_2020_179_MOESM1_ESM.pdf]

Interventions to Improve Medication Adherence in Tuberculosis Patients: A Systematic Review of Randomized Controlled Studies

Ivan S. Pradipta, Daphne Houtsma, Job F.M. van Boven, Jan-Willem C. Alffenaar, Eelko Hak

Supplementary information 1. Search strategies

1. Medline/ PubMed database:

   ((Tuberculosis[tiab] or TB[tiab] OR tuberculosis infection[tiab] OR active tuberculosis[tiab] OR latent tuberculosis[tiab] OR pulmonary tuberculosis[tiab] OR extrapulmonary tuberculosis[tiab] OR anti-tuberculosis treatment*[tiab] OR anti-tb treatment*[tiab] OR “Tuberculosis”[Mesh]) AND (Adherence[tiab] OR compliance[tiab] OR nonadherence[tiab] OR non-adherence[tiab] OR concordance[tiab] OR medication adherence[tiab] OR patient adherence[tiab] OR patient compliance[tiab] OR "Medication Adherence"[Mesh]) AND (self-administration[tiab] OR self-administered[tiab] OR DOT*[tiab] OR directly observed*[tiab] OR directly observed therapy[tiab] OR directly observed treatment[tiab] OR incentive*[tiab] OR social support*[tiab] OR patient organization*[tiab] OR education[tiab] OR adherence education[tiab] OR dose frequency[tiab] OR memory aid*[tiab] OR reminder*[tiab] OR reinforcement*[tiab] OR reminder system*[tiab] OR motivation[tiab] OR motivational tool*[tiab] OR home visit*[tiab] OR patient education[tiab] OR counseling[tiab])).

2. Cochrane database:

   #1. Tuberculosis[Mesh]
   #2. 'tuberculosis' OR 'tb' OR 'tuberculosis infection' OR 'active tuberculosis' OR 'latent tuberculosis' OR 'pulmonary tuberculosis' OR 'extra pulmonary tuberculosis' OR 'anti-tuberculosis treatment' OR 'anti-tb treatment*'
   #3. #1 AND #2
   #4. Medication Adherence [Mesh}
   #5. 'adherence' OR 'compliance' OR 'non adherence OR 'non-adherence' OR 'concordance' OR 'medication adherence' OR 'patient adherence' OR 'patient compliance'
   #6. #4 AND #5
   #7. 'self-administration' OR 'self-administered' OR 'DOT*' OR 'directly observed*' OR 'directly observed therapy' OR 'directly observed treatment' OR 'incentive*' OR 'social support*' OR 'patient organization*' OR 'education' OR 'adherence education' OR 'dose frequency' OR 'memory aid*' OR 'reminder*' OR 'reinforcement*' OR 'reminder system*' OR 'motivation' OR 'motivational tool*' OR 'home visit*' OR 'patient education' OR 'counseling'
   #8. #3 AND #6 AND #7
